# Paraoxonase 3 is involved in the multi-drug resistance of esophageal cancer

**DOI:** 10.1186/s12935-018-0657-1

**Published:** 2018-10-22

**Authors:** Dabing Huang, Yong Wang, Yifu He, Gang Wang, Wei Wang, Xinghua Han, Yubei Sun, Lin Lin, Benjie Shan, Guodong Shen, Min Cheng, Geng Bian, Xiang Fang, Shilian Hu, Yueyin Pan

**Affiliations:** 1 Department of Oncology, the First Affiliated Hospital of USTC, Division of Life Sciences and Medicine, University of Science and Technology of China, Hefei, 230001 Anhui People’s Republic of China; 2 Department of Oncology, The Affiliated Hospital of Anhui Medical University, Hefei, 230001 Anhui People’s Republic of China; 3 Department of Geriatrics, the First Affiliated Hospital of USTC, Division of Life Sciences and Medicine, University of Science and Technology of China, Hefei, 230001 Anhui People’s Republic of China; 4 Anhui Provincial Key Laboratory of Tumor Immunotherapy and Nutrition Therapy, Hefei, 230001 Anhui People’s Republic of China; 5 Gerontology Institute of Anhui Province, Hefei, 230001 Anhui People’s Republic of China

**Keywords:** Esophageal cancer, Multi-drug resistance, Methylation, PON3

## Abstract

**Background:**

Drug resistance prevents the effective treatment of cancers. DNA methylation has been found to participate in the development of cancer drug resistance.

**Methods:**

We performed the wound-healing and invasion assays to test the effect of the paraoxonase gene PON3 on esophageal cancer (EC) cells. In addition, in vivo EC-derived tumor xenografts in nude mice were generated to test the effect of PON3 on the chemoresistance of EC cells.

**Results:**

We found that PON3 is hypermethylated in drug-resistant EC cell line K150, which in-return down-regulates its expression. The following experiments by the forced changes of PON3 level in vitro and in vivo demonstrated that the PON3 expression negatively correlates with drug resistance in EC cells. Further wound-healing and invasion assays showed that PON3 suppresses the migration and invasion of EC cells.

**Conclusion:**

Our data established that PON3 is associated with the EC drug resistance, which may serve as a biomarker for the potential therapeutic treatment of EC.

**Electronic supplementary material:**

The online version of this article (10.1186/s12935-018-0657-1) contains supplementary material, which is available to authorized users.

## Background

Esophageal cancer (EC) is the eighth most common cancer worldwide, which arises from the inner lining of the esophagus [1, 2]. To date, the frequently used therapy for the treatment of EC is chemotherapy in combination with other therapeutic strategies. However, the prognosis of patients with EC remains poor and the 5-year survival rate is less than 20% [3]. This mainly results from the resistance to the commonly used drugs owing to the abuse of antibiotics [4]. There are limited salvage options for patients with refractory EC [5] and targeted therapies are not yet available. Therefore, there is an urgent need for understanding the mechanism of drug-resistance to guide the design of novel approaches for the treatment of EC.

The family of paraoxonase (PON) has three members, PON1, PON2 and PON3, that are located adjacent to each other on chromosome 7 in humans [6]. They share high levels of homology [7]. The expression level and specific activities of PON genes were found to be negatively correlated with several inflammatory disorders, such as cardiovascular diseases, type-2 diabetes, and inflammatory bowel disease [8, 9]. Moreover, PON3 expression is remarkably up-regulated in a variety of human cells, including cancer cells  [10, 11]. Recent study suggested that PON3 promotes cell proliferation and metastasis by regulating PI3K/Akt in oral squamous cell carcinoma [12]. Despite the extensive studies of PON3 in cancer cells, the roles of PON3 in EC are rarely evaluated, especially the involvement in drug resistance. In this study, we investigated the roles of PON3 in EC cells and found that PON3 is related in various biological processes in EC cells, which will give us hints for a clinical therapy of EC.

## Methods

### Cell lines and culture

The eight K30, K450, K180, K150, TE-1, K510, K140 and K410 cell lines come from our laboratory. All cell lines were cultured in RPMI1640 (Biological Industries, Israel) +10% fetal bovine serum (Invitrogen, USA) and 1% glutamine at 37 °C in 5% CO_2_.

### Bisulfite sequencing PCR (BSP) analysis

Genomic DNA was isolated by a standard phenol/chloroform purification method, verified by electrophoresis on an agarose gel, and treated by an ammonium bisulfite-based bisulfite conversion method. Then the PCR fragments from the converted DNA were sequenced and analyzed. Raw sequence data files were processed, and the area ratio (%) of C over C + T of the primary CpG dinucleotide was calculated as the % of methylation and plotted [13].

### Transient transfection assays and reagents

siRNA and scrambled (negative control, NC) sequences as well as a riboFECT CP transfection kit were supplied by Guangzhou RiboBio, China. A GFP-tagged PON3 overexpression construct (pReciever-M98) was purchased from Genecopia, Guangzhou, China (Catalog No.: EX-E0804-M98-5). Transfections of the above mentioned ribonucleic acid reagents and reporter plasmids were performed according to the manufacturer’s instructions.

### Chemoresistance profiling (IC_50_ determination)

All of the chemotherapeutic drugs used in this study were of clinical grade. To perform thiazolyl blue tetrazolium blue (MTT)-based cell proliferation assays, experimental groups of cells in the logarithmic phase of growth were seeded in triplicate in 96-well plates at a cell density of 0.5 × 10^4^/well and treated with fourfold serially diluted drugs for 72 h. Then 10 μl (5 mg/ml) of MTT salt (Sigma) was added to the corresponding wells. The cells were incubated at 37 °C for another 4 h, and the reaction was stopped by lysing the cells with 150 μl of DMSO for 5 min. The optical density was measured at 570 nm. A group that received no drug treatment was used as a reference for calculating the relative cell survival rate.

### RNA analysis

Total RNA was isolated from cells during the logarithmic phase using TRIzol (Tiangen Biotech). For mRNA analysis, a cDNA primed by an oligo-dT was constructed using a PrimeScript RT reagent kit (Tiangen Biotech). The PON3 mRNA level was quantified using duplex-qRT-PCR analysis, wherein TaqMan probes with a different fluorescence profile were used to detect β-actin (provided by Shing Gene, Shanghai, China) in a FTC-3000P PCR instrument (Funglyn Biotech). Using the 2^−ΔΔCt^ method, target gene expression levels were normalized to the β-actin expression level before the relative levels of the target genes were compared.

### Western blot protein analysis

Cells were lysed with lysis buffer (60 mM Tris–HCl [pH 6.8], 2% SDS, 20% glycerol, 0.25% bromophenol blue, and 1.25% 2-mercaptoethanol) and heated at 95 °C for 10 min before electrophoresis/Western blot analysis. The primary anti-PON3 (17422-1-AP) antibodies and anti-GAPDH (60004-1-lg) antibodies were purchased from Proteintech (San Ying Biotechnology, China) and were recognized with anti-rabbit IgG peroxidase-conjugated antibody (30000-0-AP) (San Ying Biotechnology, China), followed by an enhanced chemiluminescence reaction (Thermo Fisher Scientific, Waltham, MA, USA). Relative levels of proteins were quantified using densitometry with a Gel-Pro Analyzer (Media Cybernetics, Rockville, MD, USA). The target bands over the GAPDH band were densitometrically quantified, as indicated under each band (Additional file 1).

### Wound-healing assays

For cell motility assays, cells stably expressing si-PON3, GFP-PON3 and the corresponding NC were seeded in 24-well plates and cultured to near confluence. After 6 h of culture in RPMI1640 without FBS, a linear wound was carefully made using a sterile 10 µl pipette tip across the confluent cell monolayer, and the cell debris was removed by washing with phosphate-buffered saline. The cells were incubated in RPMI1640 plus 10% FBS, and the wounded monolayers were then photographed at 0, 8, 12 and 20 h after wounding.

### In vitro invasion assays

Cell invasion assays were performed in a 24-well plate with 8 mm pore size chamber inserts (Corning, USA). For invasion assays, 1 × 10^3^ cells stably expressing si-PON3, GFP-PON3 or NC were placed into the upper chamber in each well with the matrigel-coated membrane, which was diluted in serum-free culture medium. In the assay, cells were suspended in 100 µl of RPMI1640 without FBS when they were seeded into the upper chamber. In the lower chamber, 500 µl of RPMI1640 supplemented with 10% FBS was added. After incubation for 36 h at 37 °C and 5% CO_2_, the membrane inserts were removed from the plate, and non-invading cells were removed with cotton swab from the upper surface of the membrane. Cells that moved to the bottom surface of the chamber were stained with 0.1% crystal violet for 30 min. The cells were then imaged and counted in at least 5 random fields using a CKX41 inverted microscope (Olympus, Japan). The assays were conducted in three independent times.

### Signaling pathway analysis

The reporter construct encodes the firefly luciferase reporter gene under the control of a basal promoter element (TATA box) joined to tandem repeats of a specific transcriptional response element. The cells were transfected in triplicate with each firefly luciferase reporter construct in combination with the Renilla luciferase-based control construct using the riboFECT CP transfection reagent, and both the luciferase activities were measured in the cell extracts 24 h after transfection. The luciferase activities (luciferase unit) of the pathway reporter relative to those of the negative control in the transfected cells were calculated as a measurement of the pathway activity.

### In vivo studies

Animal experiments were performed in accordance with the National Institutes of Health Guide for the Care and Use of Laboratory Animals. Male BALB/c nude mice between 3 and 4 weeks old were used for this study [14]. K510 cells were embedded in BD Matrigel Matrix (Becton, USA) and subcutaneously injected into two sites on the back of each mouse as follows: 1.0 × 10^7^ cells/site for K510 into 2 sites/mouse, with 6 mice. Ten days after cell injection, all of the tumors were intratumorally injected with 2 nM NC/si-PON3 every 2 days. Ten days later, after four cell injections, three mice intraperitoneally received DDP (75 μg/mouse) once every other day. The remaining three mice in each group received PBS as a mock treatment control. The mice were euthanized on day 30 after four drug injections, and their tumors were weighed and imaged. Tumor weight was described as the mean ± S.D. The expression levels of PON3 and Ki67 proteins were measured using immunochemical analysis on 5 μm sections of formalin-fixed, paraffin-embedded tumor xenografts in nude mice. The antigens were retrieved by pre-treating the de-waxed sections in a microwave oven at 750 Watts for 5 min in citrate buffer (pH 6) processed with a Super Sensitive Link-Labeled Detection System (Biogenex, Menarini, Florence, Italy), and the slides were developed using 3-amino-9-ethylcarbazole (Dako, Milan, Italy) as a chromogenic substrate. After the slides were counterstained with Mayer’s hematoxylin (Invitrogen), they were mounted in an aqueous mounting medium (Glycergel, Dako). Images were captured using a Leica DM 4000B microscope (Wetzlar, Germany), the relative level of each protein was calculated using Leica software (Wetzlar, Germany), and the percentage of the mock over the chemotherapeutically treated tumors was calculated and plotted.

### Statistical analysis

All of the results are represented as the mean ± standard deviation (SD) of three independent experiments. Two-tailed Student’s *t*-test, one-way analysis of variance or Mann–Whitney U test was used to calculate statistical significance. All of the statistical analyses were performed with Microsoft Excel 2010 (Microsoft, Redmond, WA). A p-value of less than 0.05 was designated statistically significant.

## Results

### PON3 is hypermethylated in drug-resistant esophageal cancer cell line K150

**Fig. 1:**
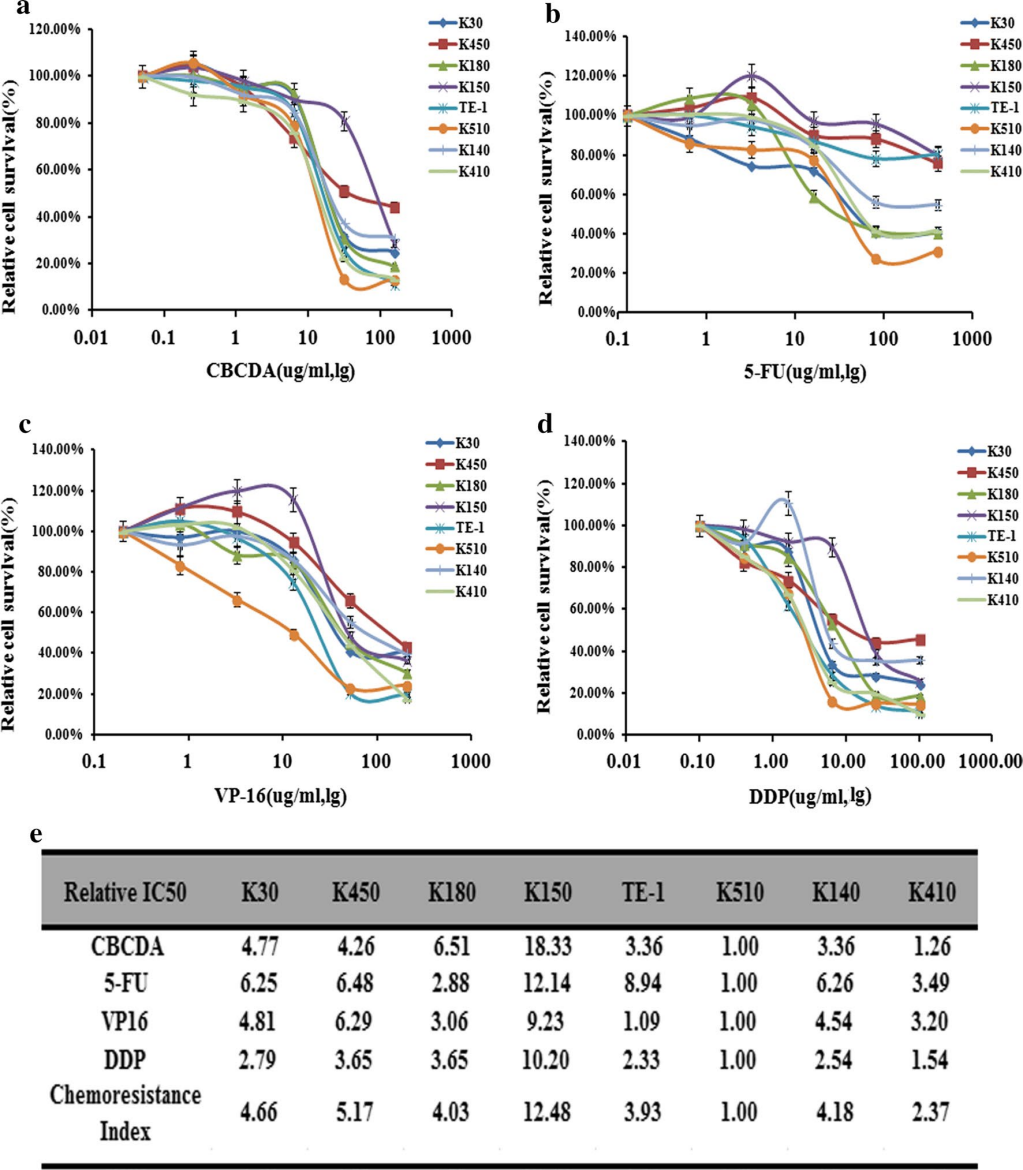
Drug resistance profiling of eight esophageal cancer cell lines. **a**–**d** IC_50_ values of the four indicated chemotherapeutics for eight esophageal cancer cell lines. The cell survival rates were calculated as percentages relative to the mock treatment and plotted against lg µg/ml of drug. **e** The IC_50_ (-fold) values relative to those of the most sensitive cell cine (K510) are presented in the table

To profile the drug resistance ability of the eight common EC cell lines (K30, K450, K180, K150, TE-1, K510, K140 and K410), we performed the IC_50_ profiling against the four drugs CBCDA, 5-FU, VP-16 and DDP, which are frequently used for EC therapy. As shown in Fig. 1, the drug resistance index revealed that K510 is the most multi-drug sensitive cell lines, with the lowest IC_50_ values against all the four drugs. In contrast, K150 is the most drug-resistant cell lines with relative drug resistance index of 12.48 (Fig. 1).

**Fig. 2:**
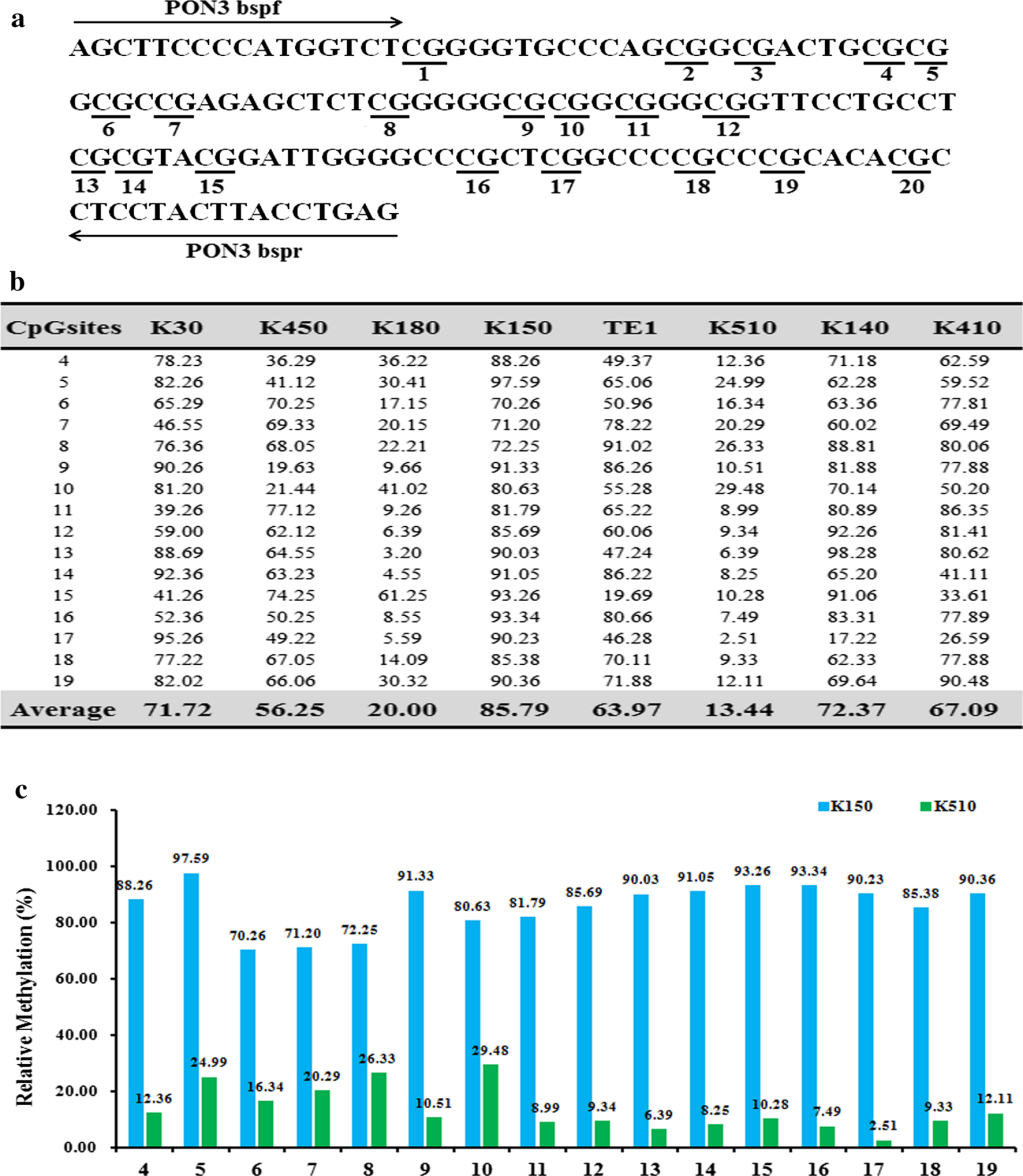
Differential methylation of the PON3 gene in eight esophageal cancer cell lines. **a** BSP primers and CpG dinucleotides of PON3 are shown. **b** Relative methylation levels (fold) of PON3 in eight esophageal cancer cell lines. **c** Methylation percentage at each CpG site in the K150 and K510 cells

To find the insight that governs the drug-resistance of EC, we tested the expression pattern of the PON3 gene, which was previously reported to involve in drug-resistance [9]. First, we detected the methylation status of the PON3 promoter region in the eight EC cells by Bisulfite Sequencing PCR (BSP) assay. The results showed that 16 CpG sites among the total 20 CpG sites were methylated at varying ratios (Fig. 2). The average methylation ratio of the PON3 gene in K150 cells is approximately 6.4-folds higher than that in K510 cells (85.79:13.44, Fig. 2b). The results suggested that PON3 is hypermethylated in the drug-resistant EC cell line K150.

### The PON3 expression negatively correlates with drug resistance in EC cells

**Fig. 3:**
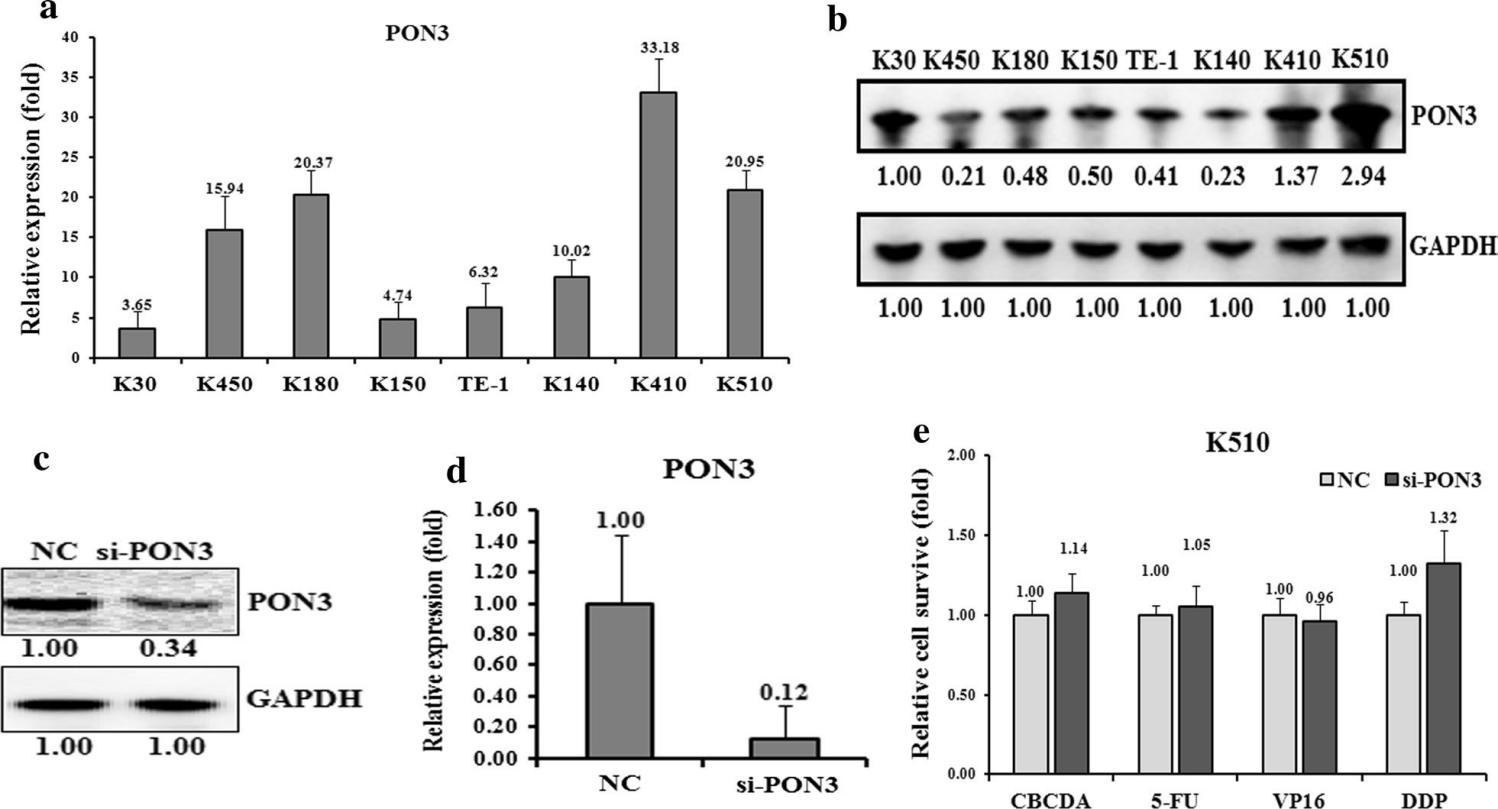
Effects of a forced reversal of the PON3 levels on the drug resistance of K510 cells. The levels of PON3 mRNA (**a**) and protein level (**b**) determined by qRT-PCR and western blot analysis in the eight esophageal cancer cell lines. The levels of protein level (**c**) PON3 and mRNA (**d**) determined by western blot and qRT-PCR analysis in the si-PON3-transfected versus the NC-transfected K510. The cell death triggered by an IC_50_ dose of four drugs in K510 cells transfected with the si-PON3-transfected versus the negative control (NC) assayed 72 h after treatment with the IC_50_ dose of drugs (**e**)

To determine whether the hypermethylation may affect the expression of PON3 in EC cells, we detected the expression levels of PON3 in the eight EC cell lines. The qRT-PCR assay revealed that the PON3 mRNA level is relatively lower in the drug-resistant cell lines K30 and K150, compared to the drug-sensitive cell lines K410 and K510 (Fig. 3a). In agreement with the mRNA level, the western blot assays also suggested that PON3 protein level is much lower in the drug-resistant cell lines K450, K140 and K150, but higher in the drug-sensitive cell lines K510 and K410 (Fig. 3b).

**Fig. 4:**
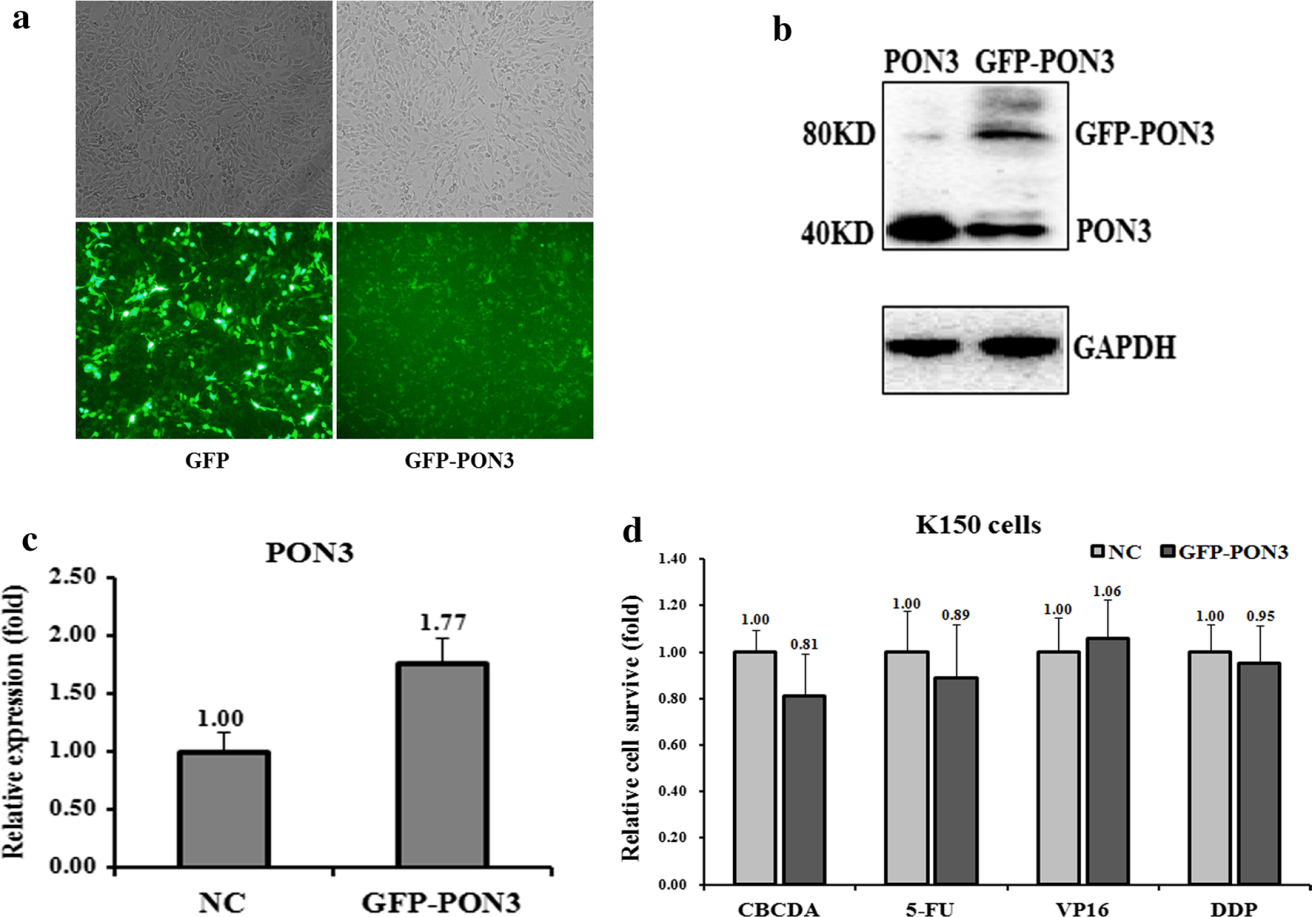
Effects of a forced reversal of the PON3 levels on the drug resistance of K150 cells. Representative areas of K150 cells transfected with GFP-PON3 ectopic expression construct were shown and GFP was used as a negative control (**a**). PON3 protein (**b**) and mRNA (**c**) level determined by western blot and qRT-PCR analysis in the GFP-tagged overexpression construct-transfected versus the NC-transfected K150. The cell death triggered by an IC_50_ dose of four drugs in K150 cells transfected with the GFP-PON3-transfected versus the negative control (NC) assayed 72 h after treatment with the IC_50_ dose of drugs (**d**)

Next, we transfected si-PON3 to down-regulate the PON3 level in K510 cells and tested the drug-resistance ability against the four drugs. Indeed, transfection of si-PON3 decreased the level of PON3 at both protein and mRNA levels, which are only 34% and 12%, respectively, of the control cells (Fig. 3c, d). Following the changes of the PON3 level in K510 cells, the cell death triggered by all four drugs was- reduced, except that against VP16 (Fig. 3e), indicating an increased drug-resistance capability. Conversely, we over-expressed GFP-tagged PON3 in K150 cells to further test the drug-resistance effect (Fig. 4a). The PON3 protein level was up-regulated by 1.77-folds following the over-expression of GFP-PON3 (Fig. 4b, c). As expected, the drug resistance ability was somewhat decreased, except that against VP16 (Fig. 4d). All these results suggest that PON3 is negatively correlated with the drug-resistance of EC cells.

### PON3 suppresses the migration and invasion of EC cells in vitro

**Fig. 5:**
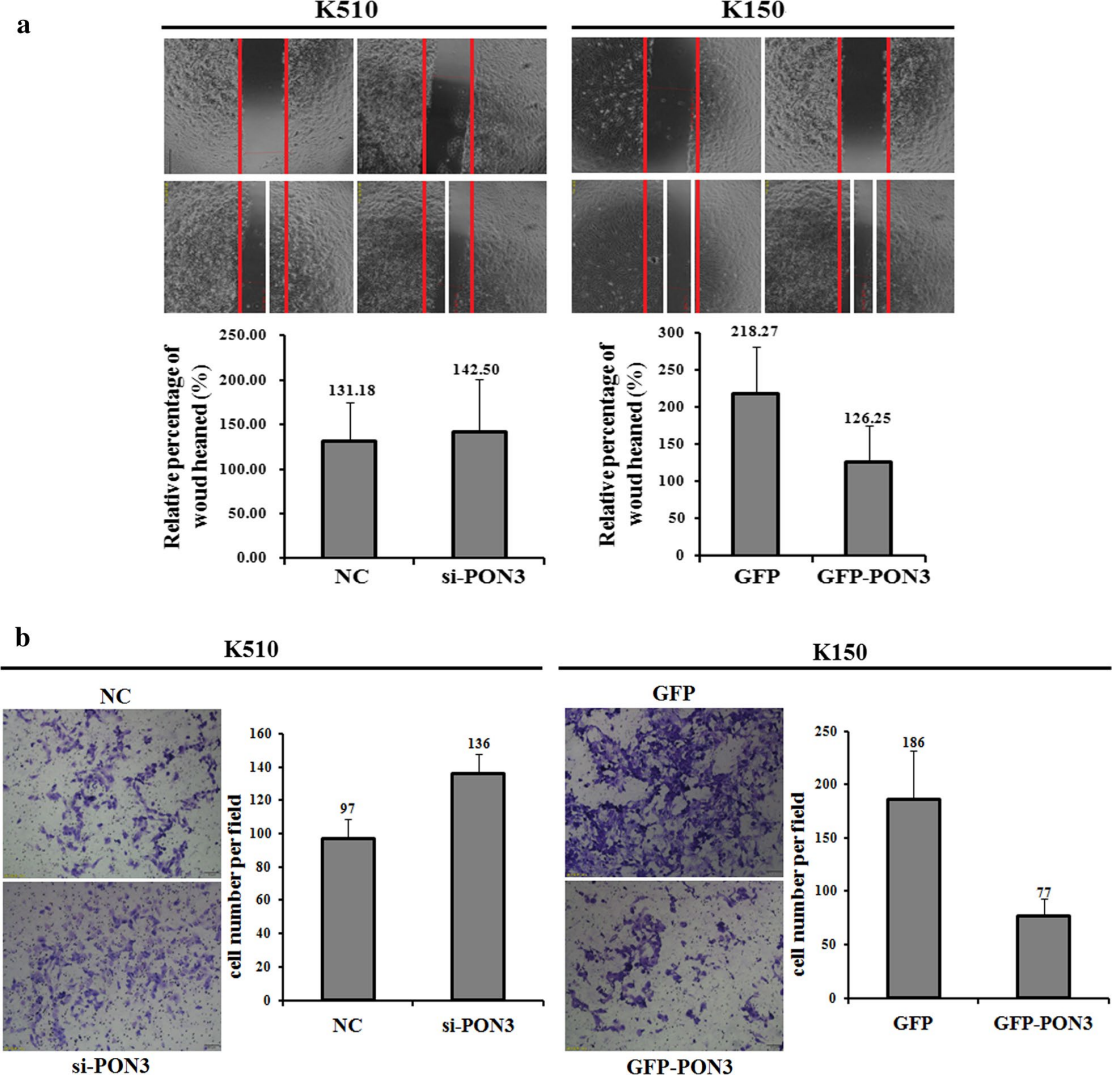
PON3 expression level affecting cell migration and invasion. Wound-healing assays that determine the migration ability of K510 and K150 cells were performed with transient expression of the si-PON3, GFP-PON3 and corresponding negative control (NC), respectively (**a**). Invasion assays that determine the invasive ability of K510 and K150 cells were performed with transient expression of the si-PON3, GFP-PON3 and corresponding negative control (NC), respectively (**b**). The data are representative of three independent experiments

The differential methylation state as well as the different expression level of PON3 in the EC cell lines indicate their potential roles in the metastasis of EC. We then compared the migration and invasion capability of K510 and K150 cells using the wound-healing and matrigel invasion assays, respectively. Compared to the control cells, transfection of si-PON3 into K510 cells significantly increased the migration capability, whereas transfection of GFP-PON3 into K150 cells largely decreased the migration capability (Fig. 5a). The results suggest that the PON3 level negatively correlates with the migration of EC cells. Similar results are also found for the invasion assays, as revealed by the transfection of either si-PON3 into K510 cells, or GFP-PON3 into K150 cells (Fig. 5b). Taken together, PON3 might act as a negative regulator of both migration and invasion of EC cells.

**Fig. 6:**
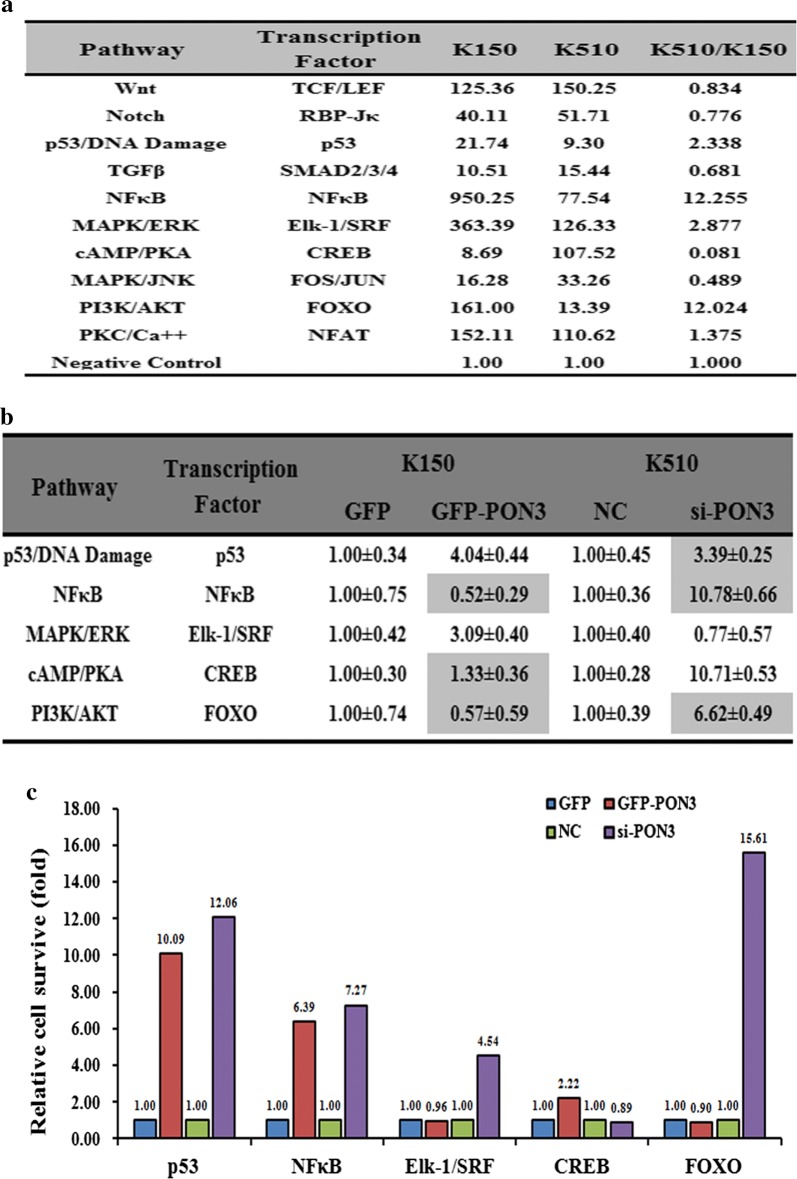
The effects of the forced reversal of PON3 levels on the activity of the signaling pathways in K150 versus K510 cells. The activities of the ten pathways in K150 versus K510 cells (**a**). The relative pathway activities in the PON3 siRNA- and GFP-PON3 versus the corresponding NC, which were transfected in K510 and K150 cells, respectively (**b**). The expression ratio of the five transcription Factors in the PON3 siRNA- and GFP-PON3 versus the corresponding NC- transfected in K510 and K150 cells, respectively (**c**)

To further understand the underlying molecular mechanisms of EC drug resistance, we measured the activities of the ten classical signaling pathways in both K510 and K150 cells. Among the ten pathways, the activities of five pathways differed by more than two-folds in K510 and K150 cells, suggesting that they might play a role in EC drug resistance. Among them, four pathways, p53/DNA damage, NF-кB, MAPK/ERK and PI3K/AKT showed higher activities in K510 cells, whereas cAMP/PKA showed higher activities in K150 cells (Fig. 6a). We then determined which of the five pathways were also affected by forced changes of the PON3 level in both K150 and K510 cells. As shown in Figure 6B and 6C, upon the repression of PON3 level by si-PON3 in K510 cells, the activities of p53/DNA damage, NF-кB, and PI3K/AKT were elevated, which correlate well with the negative regulation of these pathways by PON3 in K510 cells (Fig. 6c). We also transfected GFP-PON3 into K150 cells and measured the activities of these five pathways in K150 cells. Following the increase of the PON3 level, the NF-кB and PI3K/AKT pathways were repressed (Fig. 6c), which is in agreement with the forced changes of PON3 level in K510 cells upon transfection of si-PON3. Overall, we propose that the NF-кB and PI3K/AKT pathways involve in the EC drug resistance mediated by the PON3 gene.

### PON3 inhibits both the growth and DDP drug resistance of K510-derived tumor xenografts in nude mice

**Fig. 7:**
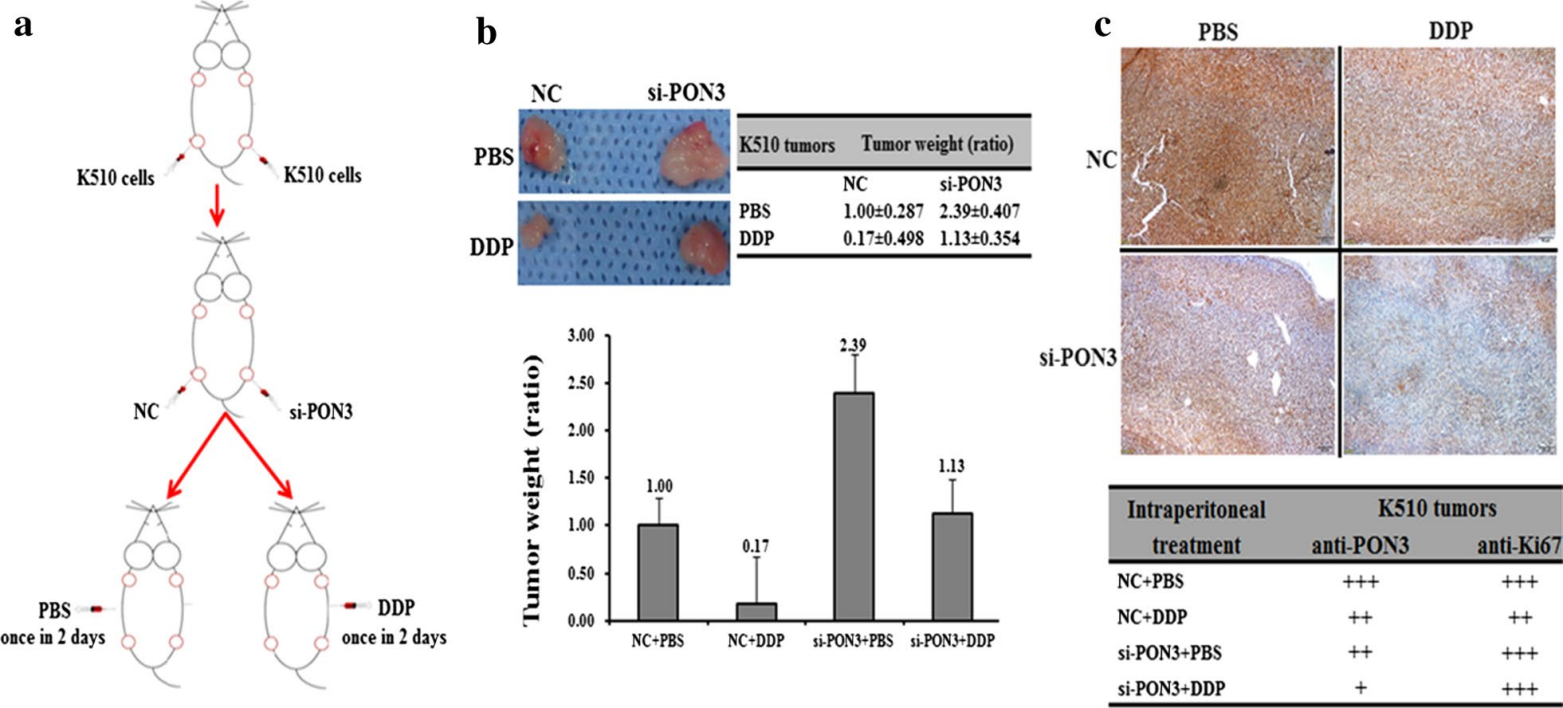
Effect of PON3 on the in vivo growth and DDP drug resistance of K510-derived xenografts in nude mice. **a** Experimental scheme: K510 cells were subcutaneously injected at two points on the back of each nude mouse, with 2 sites/mouse, 6 mice for K510. From the 15th day after cell injection, all six K150-generated tumors on the left back of the nude mice were intratumorally injected with 2nM si-PON3, and the six right back sites were injected with 2 nM Mock; this process was repeated four times within 3 days. From the 28th day after cell injection, 3 K510 mice received DDP (2.5 mg/kg) intraperitoneally once every 3 days, for a total of 4 injections over 12 days. The remaining 3 mice received PBS as a mock treatment control. **b** Image of representative tumors on the day of 45, and the mean ± SD of the tumor weight of the tumor for the same treatment was calculated, plotted and summarized. **c** The protein levels of PON3 and Ki67 in each group were determined by immunostaining and summarized in the table (Magnification: ×200)

To investigate the in vivo effect of PON3 on EC drug resistance, we generated a K510-derived tumor model in nude mice (Fig. 7a). Upon transfection of si-PON3, K510-derived tumors were approximately 2.4-folds heavier than the control cells, suggesting that PON3 inhibits tumor growth in vivo. In addition, after an intraperitoneal injection of DDP, the K510 tumors were much smaller than the control cells with the injection of PBS (Fig. 7b). Furthermore, the tumor weight for the si-PON3/DDP K510 mice was heavier than that in the DDP K510 mice (Fig. 7b). These results clearly indicated that PON3 inhibits both the growth and DDP drug resistance of K510-derived tumor xenografts in nude mice.

Further confirmation of the PON3 role in the DDP resistance of EC came from the immunohistological analysis of PON3 and Ki67 (an indicator of tumor cell proliferation) in the tumor sections of the DDP-treated versus PBS-treated mice (Fig. 7c). The intratumoral injection of si-PON3 into K510 indeed led to the decrease of the PON3 level in the tumor sections (Fig. 7c), which further confirmed that PON3 has a positive effect on both the growth and drug resistance of EC cell-derived tumor xenografts in nude mice

## Discussion

Accumulating evidences have been shown that DNA methylation play important roles in drug resistance of cancers, which prevents the effective treatment of cancers [15]. Altered DNA methylation patterns can influence the expression of genes [16]. Recent study on the profiling of gene-specific methylation levels in EC provides a useful approach for investigating the individual hypermethylated gene in EC [17]. Despite extensive studies revealed that methylation is a modulator of cancer, the understanding of DNA methylation on the effect of EC remains limited. In our study, we identified that the promoter region of PON3 is hypermethylated in drug resistant EC cell lines. The hypermethylation of PON3 in return down-regulates its expression in drug resistant EC cells. Furthermore, we showed that the PON3 level is negatively correlated with the drug-resistance of EC cells, and thus suppresses the EC drug resistance. In vivo experiments also found that PON3 inhibits tumor growth in nude mice. All these findings made us to propose that PON3 might be a tumor suppressor, considering its high methylation in the promoter region and low expression level in multiple cancers.

Paraoxonase 3 (PON3) belongs to the paraoxonase family that helps in preventing oxidative stress and anti-inflammatory [18]. This gene also involves in other diseases including cancer [19, 20]. PON3 gene has a high expression level in cancer tissues of the lung, liver and colon [11]. Previous studies also showed that PON3 is hypermethylated in colorectal cancer [9] and chordomas [21]. Notably, the genome-wide DNA methylation analysis identified that several genes, including PON3, are aberrantly methylated in the high-grade non-muscle invasive bladder cancer [22]. These findings suggest that epigenetic modifications are usually associated with the development and/or progression of different type of tumors [13, 23]. In accordance with previous studies, we identified that the promoter region of PON3 is hypermethylated in EC cancer. The hypermethylation of PON3 may serve as a marker of poor prognosis in human EC. Furthermore, the expression of PON3 negatively correlates with drug resistance in EC cells, and thus appears to act a biomarker for the drug resistance of EC cells. The study may provide a new potential therapeutic target in the treatment of EC. However, the detailed mechanism for the PON3-regulated drug resistance in EC cells remains to be clarified.

## Conclusions

In this work, we identified that PON3 is associated with the multi-drug resistance of EC cancer. Our findings suggest that PON3 may serve as a biomarker for the potential therapeutic treatment of EC.

## Additional file

**Additional file 1.** The full-length gels of the western analyses used in the manuscript.

## Abbreviations

EC: esophageal cancer
BSP: bisulfite sequencing PCR
CBCDA: carboplatin
5-FU: 5-fluorouracil
VP-16: etoposide phosphate
DDP: cisplatin
PON3: paraoxonase 3
