# Supplementary material for: Paraoxonase 3 is involved in the multi-drug resistance of esophageal cancer
Source: Cancer Cell Int. 2018 Oct 22;18:168. doi: 10.1186/s12935-018-0657-1 (PMC6198441; doi:10.1186/s12935-018-0657-1)
Supplement: Supplementary file 1 — Additional file 1. The full-length gels of the western analyses used in the manuscript. [file 12935_2018_657_MOESM1_ESM.pdf]

**Paraoxonase 3 is involved in the multi-drug resistance of esophageal cancer**

Dabing Huang[1, 2, 3,4*] , Yong Wang[1*] , Yifu He[1], Gang Wang[1], Wei Wang[1], Xinghua Han[1], Yubei Sun[1], Lin Lin[1] , Benjie Shan[1], Guodong Shen[3,4], Min Cheng[3,4], Geng Bian[2,3,4] , Xiang Fang[2,3,4] , Shilian Hu[2,3,4 §], Yueyin Pan[1 §]

[§]Co-corresponding authors.

*Co-first authors.

**E-mail addresses:**

DBH: hdabing68@163.com

YW: chwangyong@126.com

YFH: heyifu@csco.org.cn

GW: wanggang829@126.com

WW: wangwei20189@163.com

XHH: hanxinghuaslyy@163.com

YBS: sunyubeislyy@163.com

BJS: danbenjie@163.com

LL: lin131521@126.com

GDS: gdshen@ustc.edu.cn

MC: chengmin@ustc.edu.cn

GB: biangeng2018@163.com

XF: fangxiang417@126.com

SLH: hushilianslyy@vip.126.com

YYP: pyyahslyy@163.com

The full-length gels of the western analyses used in the manuscript.

**Figure 3B**

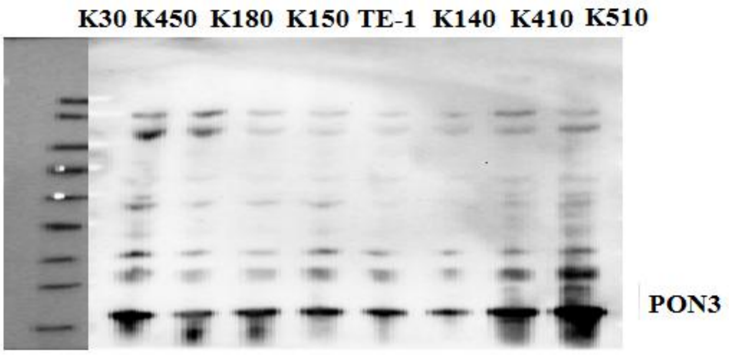


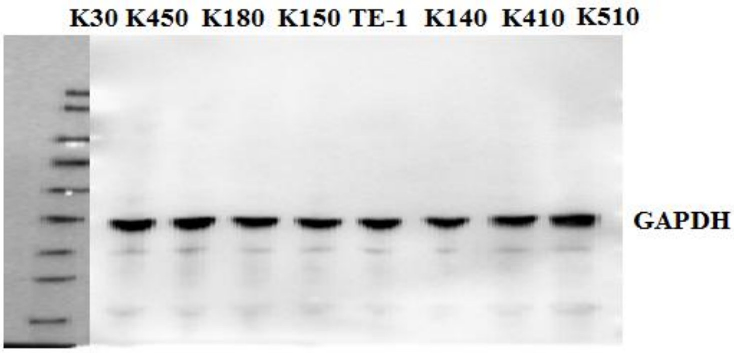


The levels of PON3 protein level determined by western blot analysis in the eight esophageal cancer cell lines

**Figure 3C**

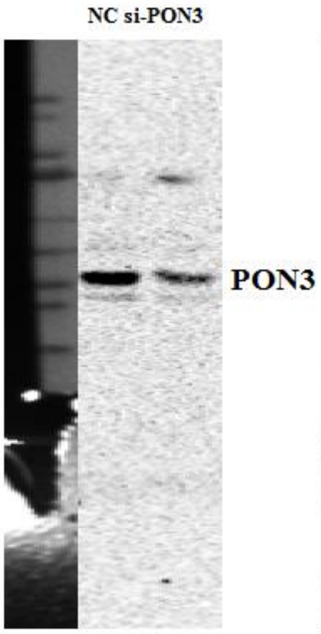


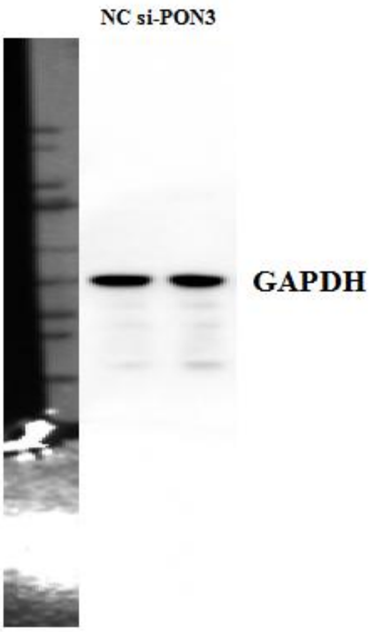


The levels of protein level (C) PON3 determined by western blot analysis in the si-PON3-transfected versus the NC-transfected K510

**Figure 4B**

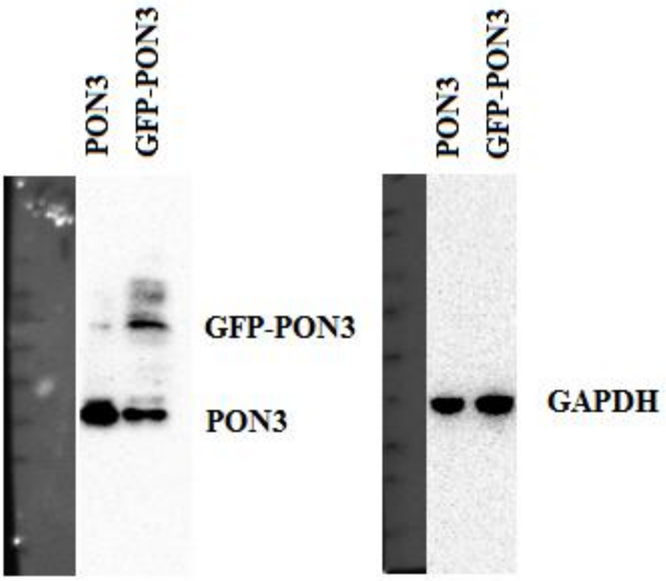


The PON3 protein level determined by western blot analysis in the GFP-tagged overexpression construct-transfected versus the NC-transfected K150
